# Suppressor of Cytokine Signaling 2 Negatively Regulates NK Cell Differentiation by Inhibiting JAK2 Activity

**DOI:** 10.1038/srep46153

**Published:** 2017-04-06

**Authors:** Won Sam Kim, Mi Jeong Kim, Dong Oh Kim, Jae-Eun Byun, Hangsak Huy, Hae Young Song, Young-Jun Park, Tae-Don Kim, Suk Ran Yoon, Eun-Ji Choi, Haiyoung Jung, Inpyo Choi

**Affiliations:** 1 Immunotherapy Convergence Research Center, Korea Research Institute of Bioscience and Biotechnology (KRIBB), Yuseong-gu, Daejeon 34141, Republic of Korea; 2 Department of Functional Genomics, University of Science and Technology, Yuseong-gu, Daejeon 34113, Republic of Korea; 3 Department of Biochemistry, School of Life Sciences, Chungbuk National University, Cheongju 28644, Republic of Korea; 4 Department of Hematology, Asan Medical Center, University of Ulsan College of Medicine, Seoul 05505, Republic of Korea

## Abstract

Suppressor of cytokine signaling (SOCS) proteins are negative regulators of cytokine responses. Although recent reports have shown regulatory roles for SOCS proteins in innate and adaptive immunity, their roles in natural killer (NK) cell development are largely unknown. Here, we show that SOCS2 is involved in NK cell development. SOCS2^−/−^ mice showed a high frequency of NK cells in the bone marrow and spleen. Knockdown of SOCS2 was associated with enhanced differentiation of NK cells *in vitro*, and the transplantation of hematopoietic stem cells (HSCs) into congenic mice resulted in enhanced differentiation in SOCS2^−/−^ HSCs. We found that SOCS2 could inhibit Janus kinase 2 (JAK2) activity and JAK2-STAT5 signaling pathways via direct interaction with JAK2. Furthermore, SOCS2^−/−^ mice showed a reduction in lung metastases and an increase in survival following melanoma challenge. Overall, our findings suggest that SOCS2 negatively regulates the development of NK cells by inhibiting JAK2 activity via direct interaction.

Natural killer (NK) cells, a member of the innate immune system, play a critical role in the immune responses against tumors, bacteria, and virus infections^1^,^2^. NK cells eliminate tumors through secretion of lytic granules and produce pro-inflammatory cytokines such as interferon-γ (IFN-γ) and tumor necrosis factor (TNF). These NK effector functions are governed by the integration of signals derived from a variety of activating and inhibitory ligands^3^.

NK cells are developed in the bone marrow (BM) and are distributed throughout the body in both lymphoid and nonlymphoid tissues. In the initial stage of development, common lymphoid progenitors (CLPs) give rise to NK precursor (pNK) cells, which express the interleukin-2/15 receptor ß (CD122), but do not express NK1.1, DX5, or Ly49. Subsequently, pNK cells become immature NK (iNK) cells, which are positive for NK1.1 and negative for DX5 and Ly49. Further differentiation leads to mature NK (mNK) cells expressing NK1.1, DX5, and Ly49. The development of NK cells is regulated by several cytokines produced by microenvironments in the fetal liver and/or bone marrow. The cytokine interleukin 15 (IL-15) is an important regulator of NK cell maturation, survival, and homeostasis^4^. IL-15 signals are delivered through trans-presentation, where membrane-bound IL-15–IL-15 receptor alpha (IL-15Rα) complexes trigger responsive cell^5^,^6^. In mice lacking IL-15 or IL-15 receptors, the number of peripheral NK cells and cytotoxic activity are severely reduced^7^. Conversely, mice transgenic for IL-15 show a dramatic increase in the number of NK cells^8^,^9^. In addition, the IL-15R signaling pathway regulates the transcriptional activity of Id2, Tox and Ets.1 for the generation of pNK cells; E4BP4, T-bet, and Eomes for the development of iNK cells; and Helios, Runx, and Blimp1 for NK cell maturation^10^.

The Janus kinase (JAK)/signal transducer and activator of transcription (STAT) signaling pathway is involved in many cellular processes, including development, differentiation, and proliferation^11^,^12^. STAT1 is known to regulate NK cell cytotoxicity and cytokine production^13^. STAT3 negatively regulates NKG2D ligand expression, and inhibiting STAT3 activation enhances NK cell cytotoxicity^14^. STAT4 is highly expressed in resting NK cells and regulates IFN-γ production upon IL-12 stimulation by activating the T-box transcription factor T-bet^15^. STAT5 is essential for NK cell development and survival by mediating IL-2 and IL-15 signaling^16^, and STAT6 has been reported to be involved in the differentiation of NK cells^17^. Although the actions of single transcription factors in the regulation of NK cell development are well known, the genetic and epigenetic regulatory networks remain largely unexplored.

The suppressor of cytokine signaling (SOCS) proteins (CIS, SOCS1-7) are negative feedback inhibitors for various cytokines signaling via the JAK-STAT pathway^18^. SOCS proteins are characterized by the presence of a Src homology 2 domain and a C-terminal conserved domain called the SOCS box^19^. SOCS1 and SOCS3 can bind directly to JAK1, JAK2 and TYK2 to inhibit JAK activity^20^,^21^. These proteins have also been demonstrated to regulate CD4^+^ T cell polarization and plasticity^22^. SOCS2 is well known to regulate the growth hormone (GH), insulin growth factor 1 (IGF-1) and prolactin signaling pathways^23^ and has also been shown to influence LPS-induced human monocyte-derived dendritic cell (DC) maturation^24^. In addition, SOCS2 regulates T helper 2 cell expansion and development of the type 2 allergic response^25^. A recent study showed that SOCS2 and SOCS3 control macrophage development and polarization by regulating cytokine and Toll-like receptor (TLR) signaling^26^. Although the role of SOCS2 has been partially studied in other immune cells, no role for SOCS2 in NK cell differentiation has yet been defined.

In the present study, we show that the loss of SOCS2 induces the increase of NK cell development *in vitro* and *in vivo*. SOCS2^−/−^ NK cells are hypersensitive to IL-15 treatment and show enhanced IL-15-driven JAK2-STAT5 signaling during NK cell development. We also show that SOCS2 inhibits JAK2 activity via direct interaction which is induced by IL-15 treatment. Furthermore, SOCS2^−/−^ NK cells show the increase of the interaction between IL-15R and JAK2 following IL-15 treatment. The increase of SOCS2^−/−^ NK cell development is reversed by JAK2 inhibitor treatment *in vitro*. Overall, our results demonstrate a novel role for SOCS2 in regulating NK cell development.

## Results

### Increase of NK cells in SOCS2^−/−^ mice

**Figure 1:**
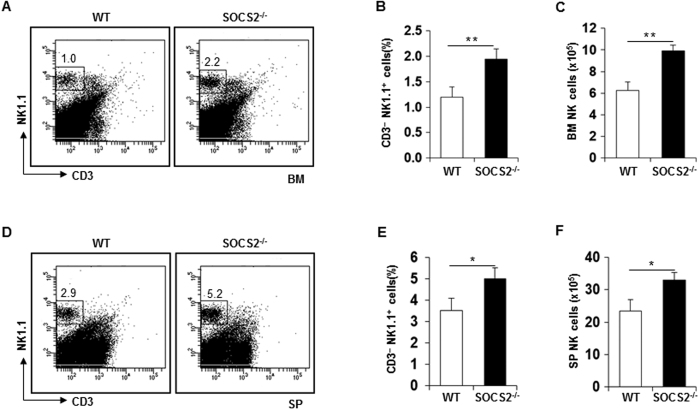
The increase of NK cells in SOCS2^−/−^ mice. (**A**) Phenotypes of CD3^−^NK1.1^+^ cells from the BM were determined by two-color flow cytometric analysis using indicated antibodies. The fluorescence intensity was analyzed from the gated lymphocyte population of WT and SOCS2^−/−^ mice. The numbers indicate the percentages of cells in the gated regions. (**B**,**C**) Bar graphs show the frequency (**B**) and the numbers of NK cells in total BM cells (**C**). The results are representative of at least two independent experiments with similar results. (**D**) Dot plots indicate the percentage of gated CD3^−^NK1.1^+^ cells in the SP. (**E**,**F**) Bar graphs show the frequency (**E**) and the numbers of NK cells in total splenocytes (**F**). n = 3–4 mice per group. *p < 0.05, **p < 0.01, and ***p < 0.001 (error bars, mean ± SD). Data are representative of at least three independent experiments.

To investigate the effects of SOCS2 on NK cell development, we analyzed the frequencies and numbers of lymphocytes in the bone marrow (BM) and spleen (SP) of wild-type (WT) or SOCS2^−/−^ mice. Previous studies have shown that there are no significant differences between T- and B-lymphoid, myeloid and erythroid cell populations in SOCS2^−/−^ mice^27^. Consistent with previous studies, no substantial differences in the population of T, B, and myeloid cells were detected between WT and SOCS2^−/−^ mice (Table S1). However, interestingly, the frequency and number of NK cells in SOCS2^−/−^ mice were increased in the BM (Fig. 1A–C) and SP (Fig. 1D–F). These data suggest that the loss of SOCS2 may be associated with enhanced NK cell development.

### SOCS2 deficiency leads to an increase in NK cell differentiation *in vitro*

**Figure 2:**
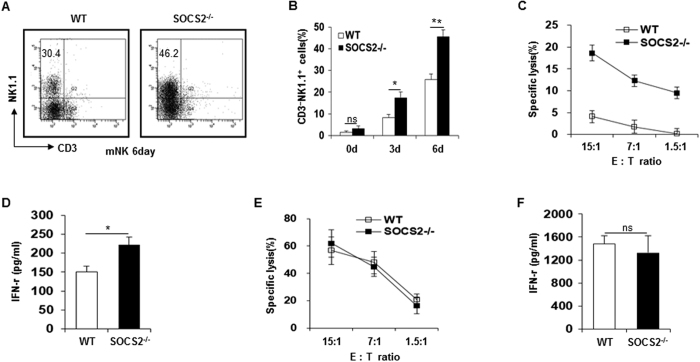
Increased NK cell differentiation of SOCS2^−/−^ HPCs *in vitro*. (**A**,**B**) NK cells were differentiated *in vitro* from HPCs of WT and SOCS2^−/−^ mice. After maturation (mNK), CD3 and NK1.1 expression were analyzed by flow cytometry. Data for days 0, 3 and 6 pooled from four experiments. *p < 0.05, **p < 0.01. (**C**) Differentiated WT and SOCS2^−/−^ NK cells were mixed at the indicated ratio with YAC-1 target cells and NK cytotoxicity was determined by a ^51^Cr release assay. (**D**) IFN- γ production of differentiated WT and SOCS2^−/−^ NK cells which are stimulated with IL-12 (20 ng/ml). *p < 0.05, **p < 0.01. (**E**) Splenic WT and SOCS2^−/−^ NK cells were cultured with IL-2 (30 ng/ml) for 16h. The cytotoxicity of NK cells was determined by a ^51^Cr release assay against YAC-1 target cells at the indicated E:T ratios. (**F**) IFN- γ production of splenic WT or SOCS2^−/−^ NK cells was quantified by ELISA. *p < 0.05, **p < 0.01. The data are representative of three independent experiments, and the error bars represent the SD of triplicates.

SOCS2 is a well-known negative regulator of the cytokine-induced signaling pathway. Because IL-15 is an essential cytokine for NK cell development^28^, we examined whether SOCS2 deficiency influenced IL-15–induced NK cell differentiation of hematopoietic progenitor cells (Lineage^−^c-Kit^+^/HPCs). HPCs obtained from WT and SOCS2^−/−^ mice were differentiated into NK cells *in vitro* and analyzed by flow cytometry. The population of CD3^−^NK1.1^+^ cells from HPCs of SOCS2^−/−^ mice was significantly increased (Fig. 2A,B). To investigate whether differentiated NK cells from SOCS2^−/−^ HPCs show NK cell activity, we performed a cytotoxicity assay using NK cells and measured the secretion of cytokines. As shown in Fig. 2C, the differentiated total cells from SOCS2^−/−^ HPCs showed an enhanced capacity to lyse target cells, which may have been due to the high frequency of NK cells among total cells. The differentiated total cells from SOCS2^−/−^ HPCs also showed markedly increased IFN-γ levels in the culture medium (Fig. 2D). To determine the effects of SOCS2^−/−^ deficiency on the activity of primary NK cells, we performed cytolytic activity assays on isolated DX5^+^ NK cells from WT and SOCS2^−/−^ mice *in vitro*. The same number of SOCS2^−/−^ NK cells had no significant effect on NK cell cytolytic activity and IFN-γ secretion when compared with WT NK cells *in vitro* (Fig. 2E,F). Therefore, these results indicate that SOCS2 serves as a negative regulator of NK cell differentiation but does not affect the functional activity of differentiated NK cells.

### The autonomous effects of SOCS2 on NK cell development

**Figure 3:**
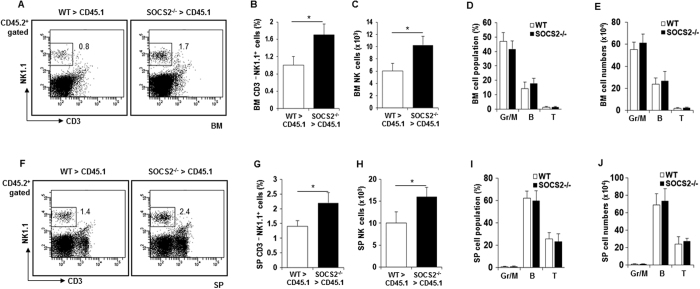
Autonomous effects of SOCS2 on NK cell development. Flow cytometric analysis of immune cells from BM or SP of chimeras at 4 months after competitive transplantation of long-term hematopoietic stem cells (LT-HSCs) (CD34^−^Flk2^−^LSK, CD45.2^+^, 5 × 10^2^) with BM cells (CD45.1^+^, 1 × 10^6^) into lethally irradiated wild-type congenic recipients (CD45.1^+^). (**A**) A representative results for NK cell frequency of CD45.2^+^ BM cells. (**B**,**C**) Percentage or numbers of NK cells (CD3^−^NK1.1^+^ in the lymphocyte gate) in CD45.2^+^ BM cells from WT and SOCS2^−/−^ HSCs. n = 5, *p < 0.05, **p < 0.01. (**D**,**E**) Percentage or numbers of granulocyte (Gr-1^+^), macrophage (CD11b^+^), T cells (CD3^+^NK1.1^−^) and B cells (B220^+^) in CD45.2^+^ BM cells derived from WT and SOCS2^−/−^ HSCs. (**F**) A representative results for NK cell frequency of CD45.2^+^ splenocytes. (**G**,**H**) Percentage or numbers of NK cells (CD3^−^NK1.1^+^) in CD45.2^+^ splenocytes from WT and SOCS2^−/−^ HSCs. (**I**,**J**) Percentage or numbers of granulocyte (Gr-1^+^), macrophage (CD11b^+^), T cells (CD3^+^NK1.1^−^) and B cells (B220^+^) in CD45.2^+^ splenocytes derived from WT and SOCS2^−/−^ HSCs. Data are from two independent experiments. Statistical significance is indicated as *p < 0.05, **p < 0.01.

Our data indicated that the loss of SOCS2 enhanced NK cell development but did not alter the functional activity of NK cells. NK cells develop from hematopoietic stem cells (HSCs) mainly in the BM^29^. Next, to avoid the environmental effect on NK cell differentiation *in vivo*, we isolated HSCs from WT and SOCS2^−/−^ mice to perform a competitive repopulation assay. We transferred HSCs from WT or SOCS2^−/−^ (CD45.2^+^) mice with competitor BM cells (CD45.1^+^) into lethally irradiated WT (CD45.1^+^) congenic mice via intravenous injection. The engraftment of WT or SOCS2^−/−^ HSCs in BM and spleen was shown no significant difference (Figure S1). However, as shown in Fig. 3A–C, the frequency and absolute number of CD3^−^NK1.1^+^ cells in BM revealed a significant increase in NK cells derived from SOCS2^−/−^ HSCs. Similarly, we observed an increase in NK cells in the spleen of the SOCS2^−/−^ chimera compared to the WT chimera (Fig. 3F–H). In contrast, we found that the frequency and total number of B cells, T cells, and granulocytes/macrophages were similar between WT and SOCS2^−/−^ chimeras in BM (Fig. 3D,E) and SP (Fig. 3I,J). These results led us to conclude that the role of SOCS2 in NK cell development was autonomous.

### Regulation of NK cell markers during NK cell differentiation *in vitro*

**Figure 4:**
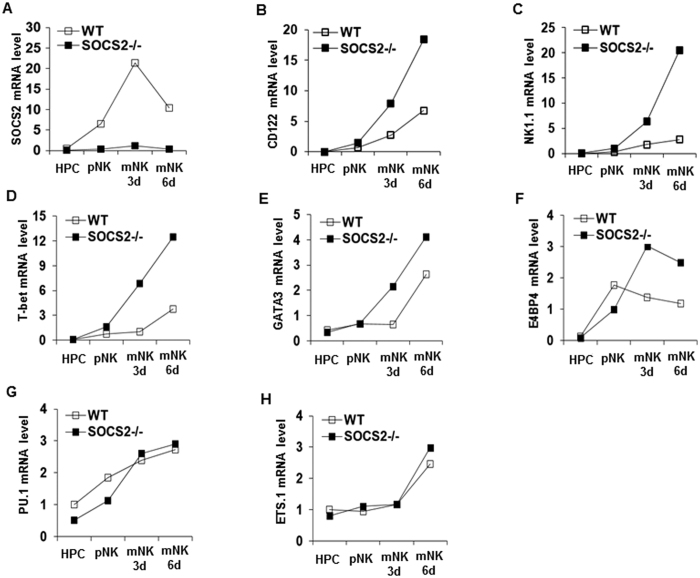
Differential induction of development-associated genes in SOCS2^−/−^ NK cells. (**A**–**H**) WT and SOCS2^−/−^ HPCs were differentiated *in vitro* with growth factors as described in Experimental Procedures. NK cell precursors were stimulated with IL-15 (30 ng/ml) for the indicated time, after which the differentiated total cells were harvested and analyzed for the relative mRNA expression of SOCS2 (**A**), NK cell development markers, CD122 (**B**), NK1.1 (**C**), or NK cell development-associated transcription factors, T-bet (**D**), GATA3 (**E**), E4BP4 (**F**), PU.1 (**G**), ETS.1 (**H**), to GAPDH by real-time PCR. The results are representative of three experiments.

To confirm the expression pattern of SOCS2 during NK cell differentiation *in vitro*, we examined the mRNA level of SOCS2 at each time point. Previous studies have shown that the expression of SOCS2 is upregulated during IL-15–mediated human NK cell differentiation^30^. When WT HPCs were differentiated *in vitro*, the expression of SOCS2 was also upregulated at the mRNA level (Fig. 4A, Fig. S2A). Next, to assess the capacity for *in vitro* differentiation, the expressions of CD122 and NK1.1 which are key markers of NK development, were compared. The expression of CD122 increased dramatically during NK cell differentiation of SOCS2^−/−^ HPCs *in vitro* (Fig. 4B, Fig. S2B). Similarly, the expression of NK1.1 also increased in SOCS2^−/−^ HPCs compared to WT HPCs (Fig. 4C, Fig. S2C). The expressions of other genes related to NK cell differentiation, including T-bet, GATA3, and E4BP4 also increased during SOCS2^−/−^ HPC differentiation (Fig. 4D–F). However, the expression of PU.1 or ETS.1 showed no differences in NK cell differentiation between WT and SOCS2^−/−^ HPCs (Fig. 4G,H, Fig. S2D).

### SOCS2 negatively regulates IL-15–induced JAK2-STAT5 activation

**Figure 5:**
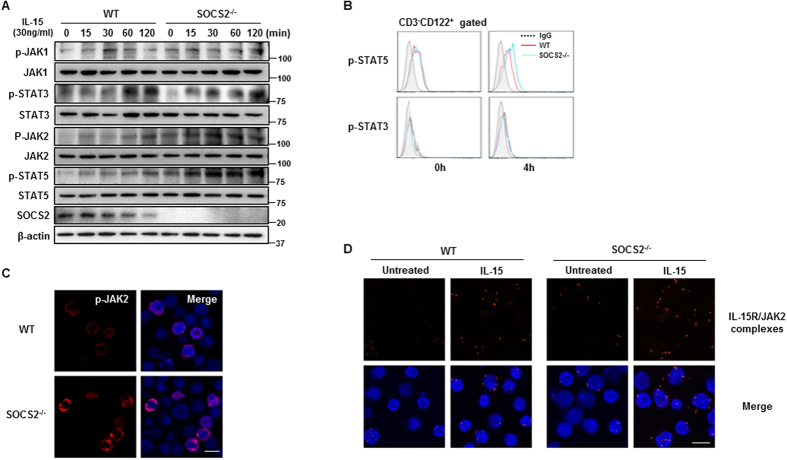
SOCS2 targets the IL-15–driven JAK2-STAT5 pathway. (**A**) Western blot analysis of WT and SOCS2^−/−^ pNK cells incubated with IL-15 (30 ng/ml) for the indicated times. Cells were lysed and detected by Western blot analysis with antibodies to the indicated phosphorylated and total proteins. (**B**) Flow cytometric analysis of phosphorylated STAT3 (bottom), STAT5 (top) in the CD122^+^CD3^−^ population of WT and SOCS2^−/−^ pNK cells. These cells were stimulated with IL-15 (30 ng/ml) for 4 hours. (**C**) The levels of phospho-JAK2 in NK cells from WT and SOCS2^−/−^ mice were determined by confocal imaging with phosphor-JAK2 antibody. (**D**) Physical interaction between IL-15R and JAK2. Splenic NK cells were treated with IL-15 (30 ng/ml) for 12 hours. Red spot represents the IL-15R-JAK2 interaction. Nuclei were stained with DAPI. Scale Bar: 20 um.

Several cytokines (IL-2, IL-12, IL-15 and IL-18) that signal via the JAK/STAT pathways are critical for NK cell development and activation^4^,^31^,^32^. Thus, we examined whether SOCS2 deficiency influenced the IL-15–mediated NK receptor signaling pathway. The phosphorylation of JAK2 and STAT5 was increased in cultured SOCS2^−/−^ pNK cells in comparison to WT cells at early time points after IL-15 stimulation. In contrast, the absence of SOCS2 had no effect on IL-15–induced JAK1-STAT3 phosphorylation (Fig. 5A). Additionally, in flow cytometric analysis, an increase of STAT5 phosphorylation was observed in IL-15–treated SOCS2^−/−^ pNK cells (Fig. 5B). Primary NK cells from SOCS2^−/−^ mice also displayed an increase in JAK2 phosphorylation following IL-15 stimulation (Fig. 5C, Fig. S3A). These results suggest that SOCS2 may target the IL-15–driven JAK2-STAT5 pathway. Previously we reported the regulation of human NK cell function via Pyk2 regulation by SOCS2. To examine the Pyk2 regulation in mouse NK cells by SOCS2, we determined the phosphorylation of Pyk2 and total Pyk2 in WT and SOCS2^−/−^ NK cells. Mouse NK cells showed similar responses with human NK cells in the regulation of Pyk2 phosphorylation by the loss of SOCS2, however, the endogenous level of Pyk2 was very low in mouse NK cells (Fig. S3A,B). However, as shown in Fig. 2E, SOCS2^−/−^ NK cells did not show increased cytotoxicity. Next, we confirmed the interaction between IL-15R and JAK2 using Duolink *in situ* proximity ligation assay (PLA). This method enabled us to monitor the subcellular localization of endogenous protein-protein interactions^33^. In SOCS2^−/−^ NK cells treated with IL-15, we found a number of strong fluorescence signals, which indicated the interaction between IL-15R and JAK2, whereas a small number of signals were detected in WT NK cells (Fig. 5D). Taken together, these results suggest that SOCS2 may regulate the IL-15R-JAK2 axis directly in NK cell development.

### SOCS2 interacts with JAK2 directly

**Figure 6:**
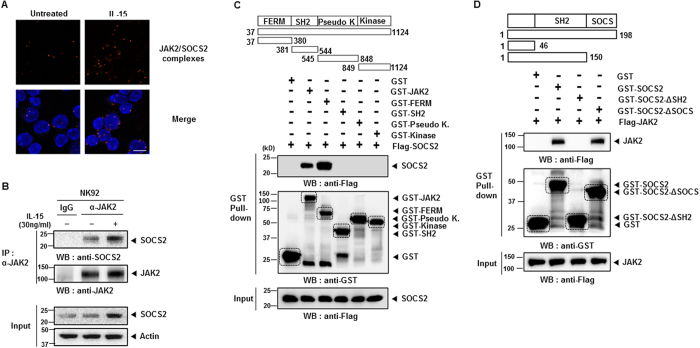
The physical interaction between SOCS2 and JAK2 in NK cells. (**A**) PLA images for physical interaction between SOCS2 and JAK2. Splenic NK cells was treated with IL-15 (30 ng/ml) for 12 hours. Each red spot represents a SOCS2-JAK2 interaction. Nuclei were stained with DAPI. Scale Bar: 20 um. (**B**) Interaction of JAK2 with SOCS2 following IL-15 treatment. NK-92 cells were treated with IL-15 (30 ng/ml) for 12 hours. Cell lysates were immunoprecipitated with JAK2 antibodies. Data are representative of two independent experiments. (**C**,**D**) Identification of the interaction domains between JAK2 and SOCS2. 293 T cells were transiently transfected with the indicated plasmids and then cell lysates were subjected to a pull-down assay with glutathione-Sepharose beads. Data are representative of three independent experiments.

SOCS proteins are known to interact with phosphorylated tyrosine motifs in target proteins via an SH2 domain^23^,^34^. Hence, we hypothesized that SOCS2 might be downregulating a target protein involved in IL-15–induced NK cell differentiation and that this regulation might be recovered by the loss of SOCS2. To verify the endogenous interaction between SOCS2 and JAK2, we examined the interaction among primary NK cells using PLA. In the primary NK cells treated with IL-15 for SOCS2 and JAK2 activation, we identified the interaction between SOCS2 and JAK2 (Fig. 6A). Furthermore, the endogenous interaction between SOCS2 and JAK2 was confirmed in NK-92 cells using co-immunoprecipitation (IP) assays (Fig. 6B). To characterize the domain necessary for the SOCS2-JAK2 interaction, we investigated the binding of SOCS2 to four domains of JAK2^11^. We found that the N-terminal FERM domain of JAK2 is important for their interaction (Fig. 6C). Next, we determined which region of SOCS2 interacted with JAK2. JAK2 precipitated full-length SOCS2 and a SOCS box deletion mutant (GST-SOCS2-∆SOCS) but not a SH2 domain-deletion mutant (GST-SOCS2-∆SH2) (Fig. 6D). Overall, these data suggest that SOCS2 is capable of regulating JAK2-STAT5 signaling pathway via direct interaction.

### Knock-down of SOCS2 induces NK cell differentiation

**Figure 7:**
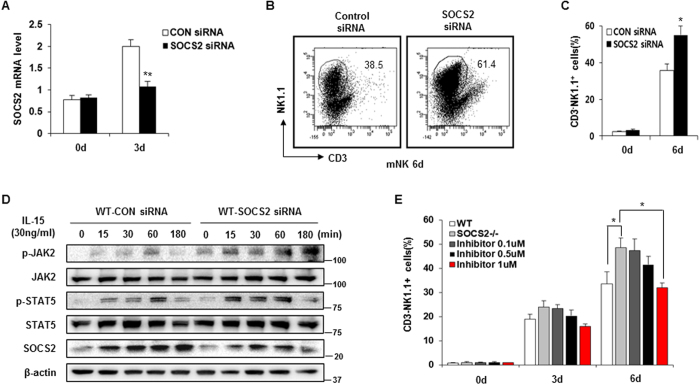
JAK2 dependent development of SOCS2 knock-down HPCs. (**A**) The expression of SOCS2 in control or SOCS2 siRNA introduced precursors which were incubated with IL-15 (30 ng/ml). (**B**,**C**) The differentiation of SOCS2 in control or SOCS2 siRNA introduced precursors which were incubated with IL-15 (30 ng/ml). The frequency of NK cells was evaluated by flow cytometry. (**D**) Western blot analysis of WT and SOCS2^−/−^ pNK cells which were incubated with IL-15 (30 ng/ml) for the indicated times. (**E**) Inhibition of SOCS2^−/−^ NK cell development by JAK2 inhibitor. Cells were pretreated with the JAK2 inhibitor for 1 h prior to the addition of IL-15 (30 ng/ml). The cells that were cultured for the indicated times were stained with anti-NK1.1 and CD3 and assayed by flow cytometry. Data are from two independent experiments. Statistical significance is indicated as *p < 0.05, **p < 0.01.

To examine whether the reduction of SOCS2 induced NK cell differentiation, we first evaluated the knock-down efficiency of SOCS2 siRNAs at the mRNA level. As shown in Fig. 7A, SOCS2 expression was significantly reduced in NK cells after 3 d. In addition, we analyzed the frequency of NK cell differentiation *in vitro* from control siRNA- or SOCS2 siRNA-treated NK cell precursors. The SOCS2 siRNA-treated NK cell precursors showed a high proportion of differentiated CD3^−^NK1.1^+^ cells (Fig. 7B,C). Subsequently, we examined the effects of SOCS2 knockdown on the IL-15–mediated NK receptor signaling pathway. We identified that knockdown of SOCS2 considerably enhanced the phosphorylation of JAK2 and STAT5 following IL-15 stimulation (Fig. 7D). Next, to confirm the role of JAK2 on NK cell differentiation, we treated Fedratinib, a JAK2 specific inhibitor. We differentiated WT or SOCS2^−/−^ HPCs to NK cells in the presence of various concentrations of the JAK2 inhibitor, and we found that the differentiation of SOCS2^−/−^ NK cells was gradually down-regulated by the JAK2 inhibitor in a dose-dependent manner (Fig. 7E). These results demonstrated that the knockdown of SOCS2 resulted in an enhancement of NK cell differentiation via the upregulation of JAK2-STAT5 signaling pathways.

### Increased tumor surveillance by NK cells in SOCS2^−/−^ mice

**Figure 8:**
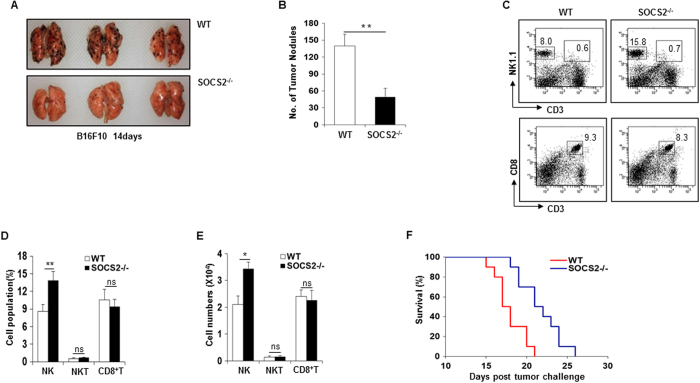
Increased tumor surveillance of NK cells in SOCS2^−/−^ mice. (**A**,**B**) Lung metastasis of WT or SOCS2^−/−^ mice was analyzed at 14 days after intravenous injection of B16F10 cells. Representative images of the lung (**A**) and the number of melanoma nodules on the surface of lung (**B**) are shown. (**C**) Representative images of flow cytometric analysis of lung-infiltrating NK, NKT, and CD8^+^ T cells are shown (n = 4). (**D**,**E**) Bar graphs show the percentage (**D**) or absolute numbers (**E**) of NK (CD3^−^NK1.1^+^) cells, NKT (CD3^+^NK1.1^+^) and CD8^+^T (CD3^+^NK1.1^−^) cells in the lungs (n = 4). (**F**) Mice survival assay. Survival rate were measured from day 0 to day 30 after melanoma injection (n = 10). The experiment shown is representative of three experiments. Statistical significance is indicates as *p < 0.05, **p < 0.01.

NK cells are well known for their tumor-suppressive role^35^,^36^. To investigate whether loss of SOCS2 affects NK cell-mediated anti-tumor activity *in vivo*, we challenged WT and SOCS2^−/−^ mice with B16F10 melanoma cells^37^. After 14 d, the experiment was terminated and lung metastases were counted. As shown Fig. 8A and B, WT mice showed extensive metastatic nodule formation in their lungs, while B16F10 metastatic nodules were largely absent in SOCS2^−/−^ mice. Next, we analyzed the infiltrated immune cells of lung at 14 days using flow cytometry. The frequency and absolute numbers of NK cells were markedly increased in SOCS2^−/−^ mice, whereas NKT cells and CD8^+^ T cells were not increased (Fig. 8C–E). To clarify the reason for increased infiltration of NK cells in the metastatic lungs, we analyzed the frequency of NK cells in the lungs of WT and SOCS2^−/−^ mice. Interestingly, the frequency of NK cells in SOCS2^−/−^ mice were increased in the lungs (Fig. S4A,B). Next, to identify NK cell migration, we transferred purified CD45.2^+^ WT or SOCS2^−/−^ NK cells into CD45.1^+^ recipients and assessed distribution of infiltrated NK cells in the lungs. The frequency of adoptively transferred NK cells migrated into the lung was not different between WT and SOCS2^−/−^ mice (Fig. S4C–E). These results imply that reduction of lung metastasis might be associated with increased number of NK cells in SOCS2^−/−^ mice. Furthermore, SOCS2^−/−^ mice survived longer than WT mice following B16F10 challenge (Fig. 8F). These data revealed that NK cells performed enhanced tumor surveillance in SOCS2^−/−^ mice.

## Discussion

Previous studies have indicated a role for SOCS2 in a variety of immune cells^22^,^24^,^26^, but no role for SOCS2 had previously been identified in NK cell differentiation. In the present study, we show that SOCS2 functions as a negative regulator of IL-15 signaling in NK cell development and that knockdown of SOCS2 results in increased activity of JAK2 and STAT5, with a corresponding increase in NK cell differentiation. Consequently, SOCS2^−/−^ mice showed enhanced tumor surveillance.

SOCS2 has primarily been shown to regulate GH^38^ and cytokine signaling and has not previously been thought to influence NK cell development. However, recent evidence suggested that SOCS2 might be involved in the regulation of NK cell development. In particular, SOCS2 expression is increased during DC maturation, and SOCS2 silencing leads to impaired maturation of DCs^24^. SOCS2 also plays an important role in regulating Th2 development of type 2 allergic responses^25^. More recently, SOCS2 was shown to control macrophage polarization during inflammation^26^. Here, we showed that IL-15–mediated NK cell differentiation was increased in SOCS2^−/−^ mice (Fig. 2A), although SOCS2 did not affect IL-15–mediated NK cell effector functions (Fig. 2E,F). However, in our previous report, SOCS2 knockdown in human NK cells led to a marked decrease of effector functions^30^. As shown in our previous report and the present report, human and mouse NK cells have shown differential responses in NK cell development and NK cell activation following the loss of SOCS2 expression and IL-15 stimulation. Basically, our protocols for NK cell development *in vitro* are different in human and mouse NK cells. As shown in our reports, human NK cells were gradually maturated for 14 days, however mouse NK cells were rapidly maturated for 7 days following IL-15 stimulation from pNKs. Human and mouse NK cells also showed differential responses on STAT5 phosphorylation following the loss of SOCS2 expression and IL-15 stimulation. In our previous report, SOCS2 knock-down did not affect IL-15-mediated STAT5 phosphorylation in human NK92 cells, but JAK2/STAT5 pathway was more activated in SOCS2^−/−^ mouse NK cells compared to WT mouse NK cells. Previously, we also have reported the regulation of human NK cell function via Pyk2 regulation by SOCS2. To examine the Pyk2 regulation in mouse NK cells by SOCS2, we determined the phosphorylation of Pyk2 and total Pyk2 in WT and SOCS2^−/−^ mouse NK cells. As shown in Figure S3, mouse NK cells showed similar responses with human NK cells in the regulation of Pyk2 phosphorylation by the loss of SOCS2, however, the endogenous level of Pyk2 expression was very low in mouse NK cells and the reduction of Pyk2 expression was dramatic in human NK cells following IL-15 stimulation but was not in mouse NK cells. Thus, this might be insufficient to regulate the activation of mouse NK cells. In Fig. 4 and Figure S2, we showed the differential expression of common transcription factors for NK development, including T-bet, GATA3, E4BP4, PU.1 and ETS-1 in response to knock-down of SOCS2 expression. Although SOCS2^−/−^ NK cell development was higher than WT, T-bet, GATA3 and E4BP4 were differentially expressed in WT and SOCS2^−/−^ NK cells but PU.1 and ETS-1 were not. Altogether, SOCS2 may regulate IL-15-mediated signaling pathways differentially in human and mouse NK cells via different partners. This differential effect of SOCS2 between human and mouse NK cells should be examined more in future studies.

Previous reports have demonstrated that SOCS2 has both positive and negative effects on GH signaling and immune response^39^,^40^. One group showed that SOCS2 could be induced by GH and could bind to the GST-fused GH receptor^41^,^42^. These studies have also shown an increase in the negative effect of SOCS2 by co-expression of the GH receptor with reporter gene assay. These findings suggest that SOCS2 might play a role in intracellular homeostasis. Thus, we hypothesized that SOCS2 might be involved in the homeostasis of IL-15–mediated NK cell differentiation via regulation of intracellular signal transduction. The SH2 domain of SOCS2 binds to a phosphorylated tyrosine residues in target proteins dependent on the SOCS box^43^. But previously, only SOCS1 and SOCS3 have been known to bind to and regulate JAK activity. SOCS1 and SOCS3 inhibit JAK activity through direct binding of the kinase inhibitory region (KIR). SOCS2 does not have a KIR region and there has been no evidence that it is able to regulate JAK activity. In our study, we describe a novel mechanism by which SOCS2 inhibits IL-15–mediated NK cell differentiation and JAK2-STAT5 activation. We showed that SOCS2 bound directly to JAK2 and this interaction was necessary for efficient regulation of JAK2-STAT5 signaling. Experiments using various mutants of SOCS2 and JAK2 demonstrated that SOCS2 binding to JAK2 was a prerequisite for IL-15 signal inhibition in NK cell development. Thus, our study provides a new molecular basis for the regulatory effect of SOCS2 on NK cell development.

The JAK-STAT signaling pathway is involved in many cellular processes, including development, differentiation, and proliferation^12^. STAT transcription factors may induce or repress transcription and have been implicated in NK cell development and function^44^. JAKs are stably associated with cytokine receptors and induce the activation of STATs upon receptor stimulation, with STAT5 being predominantly activated by IL-2, IL-7, and IL-15^45^. STAT5 is essential for NK cell development and survival mediated by IL-2 and IL-15 signaling^16^. The fact that IL15^−/−^ and IL15Rα^−/−^ mice are largely devoid of peripheral NK cells suggests that IL-15 is a dominant cytokine, regulating NK cell development and homeostasis. However, the precise role of STAT5 in this cytokine-signaling network and NK cell development remains to be determined.

In conclusion, we have shown that IL-15-induced expression of SOCS2 in NK cells restricted NK cell differentiation and that enhanced JAK2 and STAT5 phosphorylation in the absence of SOCS2 favored NK cell development. SOCS2 was shown to regulate the JAK2-STAT5 pathway via direct interaction with JAK2 and this regulation was important for NK cell development. Therefore, our results indicate a new regulatory function for SOCS2 in NK cell development.

## Materials and Methods

### Mice and Reagent

SOCS2^−/−^ mice were gifted by Dr. Nicos A. Nicola (The Walter and Eliza Hall Institute of Medical Research, Victoria 3050, Australia), and C57BL/6 mice were purchased from KoaTech (Pyeongtaek, Korea). All mice were housed under specific pathogen-free (SPF) condition and used at 10~12 weeks. All animal experiments were approved by the Institutional Animal Use and Care Committee of the Korea Research Institute of Bioscience and Biotechnology and were performed in accordance with the Guide for the Care and Use of Laboratory Animals published by the US National Institutes of Health.

### Generation of mouse bone marrow-derived NK cells

NK cell differentiation from HPCs was performed as previously described. In brief, to isolate HPCs, Lin^−^ (B cell (B220), T/NK cells (CD2), granulocytes (Gr-1), monocytes (CD11b), NK/NKT cells (NK1.1), and erythrocytes (TER-119)) cells were purified using the MACS Cell Seperation kit (Miltenyi Biotec). c-Kit^+^ cells from the Lin^−^ cells were positively selected using the CD117 (c-Kit) microbeads (Miltenyi Biotec). The purified HPCs were plated into a 24-well plate (BD) at 1 × 10^6^ cells/well and cultured for 7 d in complete RPMI1640 medium supplemented with a mixture of mouse Flt3L (50 ng/ml, Peprotech), mouse SCF (30 ng/ml, Peprotech), mouse IL-7 (0.5 ng/ml, Peprotech), Indometacin (2 ug/ml, Sigma), and gentamycin (20 ug/ml, Sigma). The cell were refreshed with the same media on day 3. To generate the mNK cells, 7 d-HPCs were maintained with IL-15 (30 ng/ml, Peprotech) for 6~7 days.

### Real-time PCR

Total RNA was extracted using the RNeasy Mini kit (Qiagen) according to the manufacturer’s instructions. Total RNA (1 ug) was reverse-transcribed using cDNA synthesis kit (Toyobo), and real-time PCR was performed in a Dice TP 800 Thermal Cyclear with SYBR Premix Ex Tag (Takara Bio). All the measurements were normalized to the housekeeping genes GAPDH.

The primer sequences were as follows: ID2, 5′-GCAAAAGAAAGAGAAAGTAAGA-3′ and 5′-GAACACGGACATCAGCATC-3′; GATA-3, 5′-TCACACACTCCCTGCCTTCT-3′ and 5′-CACCCCATTACCACCTATCC-3′; T-bet, 5′-CCAAGACCACATCCACAAAC-3′ and 5′-CAACCAGCACCAGACAGAGA-3′; E4BP4, 5′- CGGAAGTTGCATCTCAGTCA-3′ and 5′-GCAAAGCTCTCCAACTCCAC-3′; PU.1, 5′-GGTCATCTTCTTGCGGTTCT-3′ and 5′-CCTTCCAGTTCTCGTCCA-3′; ETS-1, 5′-CAGCCTCAAGATCATCAGCA-3′ and 5′-GTCTTCGGGTGGCAGTGAT-3′; GAPDH, 5′-AACTTTGGCATTGTGGAAGG-3′ and 5′-GGATGCAGGGATGATGTTCT-3′.

### Western blot analysis

Cells were lysed with RIPA buffer (50 mM Tris–HCl, pH 7.5, 1% Nonidet P-40, 1 mM EDTA, 1 mM phenylmethylsulfonyl fluoride, l g/ml leupeptin, 1 mM sodium vanadate, and 150 mM NaCl). The cell lysate was resolved on a 8-12% SDS-PAGE gels and transferred to PVDF membrane (Millipore, Bedford, MA). The membrane was probed with primary antibodies specific to the following molecules: SOCS2, JAK2, p-JAK2, JAK3, p-JAK3, STAT3, p-STAT3, STAT5, p-STAT5 (Cell Signaling Technology); and β-actin (Santa Cruz). After incubation with peroxidase-conjugated anti-rabbit or anti-mouse IgG (Jackson ImmunoResearch), the signals were detected using SuperSignal West Pico Chemiluminescent Substrate (Pierce).

### siRNA nucleofection and transfection

Nucleofection of NK cell precursors (NKPs) were performed using the Amaxa Mouse T cell Nucleofector Kit (program X-01). According to the manufacturer’s instruction, the NKPs were collected on day 7. The required numbers of cells (1 × 10^6^ cells per sample) were resuspended in 100 ul mouse nucleofector solution. 50 nM siRNA was added, and the mixed samples were transferred into certified cuvette and added 500 ul pre-warmed culture medium. The transfected cells were transferred into wells of 12-well plates, and analyzed after 48 hours. For transient expression, NKPs were transfected with 2.5 ug of the empty vector or 50 nM siRNA using Dharmalfect Transfection Reagents (Thermo Scientific), according to manufacturer’s instructions. The nontarget control siRNA, the SOCS2-specific siRNA were purchased from Dharmacon (Chicago, IL).

### Flow cytometric analysis

The following antibodies were obtained from BD Biosciences: CD3 (145-2C11), NK1.1 (PK136), CD122 (5H4), p-STAT3 (Tyr705), p-STAT5(Tyr694), CD45.2 (104), Ly-6G and Ly-6C (RB6-8C5), CD11b (D12), B220 (30-F11), CD8 (SK1). For flow cytometry, cultured cells were washed with ice-cold PBS, and then incubated with mAbs for 20 min at 4 °C, and then washed out. For intracellular staining, cells were fixed and rendered permeable using the Fix and Perm kit (BD Biosciences), according to the manufacturer’s instructions. Data were analyzed using FACSCalibur (BD Biosciences).

### Plasmids and transient transfection

The pEBG vector encoding full-length SOCS2 and deletion plasmids have been previously described^30^. To generate GST-JAK2, the cDNA encoding human JAK2 was amplified from an NK-92 cDNA library by PCR with specific primers. The PCR product was cloned into the ClaI and NotI sites of the pEBG vector. The deletion mutants of GST-JAK2, FERM-, SH2-like-, pseudokinase- and the kinase domains, were constructed by self-ligation of ClaI/NotI-digested fragment from GST-JAK2 full length, respectively. To generate Flag-JAK2 the cDNA encoding human JAK2 was amplified from an NK-92 cDNA library by PCR with specific primers. The PCR product was cloned into the EcoRV and SmaI sites of the Flag vector. To generate Flag-SOCS2 the cDNA encoding human SOCS2 was amplified from an NK-92 cDNA library by PCR with specific primers. The PCR product was cloned into the EcoRI and SalI sites of the Flag vector. For transient expression, HEK293T cells were transfected with the vectors indicated in Fig. 5 using Plus/Lipofectamine reagent (Invitrogen) according to the manufacturer’s instructions.

### Immunofluorescence microscopy

To primary NK cells preparation, we used FACSAria Cell Sorter (BD Biosciences). NK cells were harvested 1 h after IL-15 (30 ng/ml) treatment. Cells were plated on round glass cover slips in 24-well plates for 10 min. The samples were washed with cold PBS and fixed for 20 min at room temperature with 4% paraformaldehyde in PBS. The cells were then permeabilized with 0.2% Triton X-100 in PBS for 15 min at 4 °C and incubated for 2 h at room temperature with either anti-phospho JAK2 antibody. After three washes with PBS, the cells were incubated with Alexa Fluor 546-conjugated goat-anti mouse IgG (Invitrogen) for 1 h at room temperature and then washed again with PBS. The images were captured using a LSM510 confocal microscope (Carl Zeiss, Gottingen, Germany).

### Immunoprecipitation and GST pull down

Immunoprecipitation of endogenous SOCS2 with endogenous JAK2 was analyzed in NK-92 cells. Sixteen hours after IL-15 (30 ng/ml) treatment, the cells were harvested and lysed in lysis buffer containing 0.5% Triton X-100, 150 mM NaCl, 10% glycerol, 20 mM HEPES (pH 7.2), and 1x protease inhibitor mixture (Calbiochem, San Diego, CA). The cell lysates were then incubated with different Abs for 2–3 h at 4 °C. The Ag-Ab complexes were precipitated by incubation at 4 °C for 3 h with protein G-conjugated agarose (Roche). The immunoprecipitated complexes were washed and analyzed by Western blot. For the GST-pull down assay, 293T cells were transfected with various combinations of expression vectors, as indicated in the text. After lysis, cell lysates were incubated with glutathione Sepharose 4B (GE Healthcare, Piscataway, NJ) for 3 h at 4 °C. The glutathione Sepharose 4B pellets were washed and analyzed by Western blot.

### Statistical analysis

The data are expressed as the mean ± SD of n determinations and statistical significance was determined using Student’s *t*-tests. *P < 0.05, **P < 0.01, ***P < 0.001.

## Additional Information

**How to cite this article:** Sam Kim, W. *et al*. Suppressor of Cytokine Signaling 2 Negatively Regulates NK Cell Differentiation by Inhibiting JAK2 Activity. *Sci. Rep.* **7**, 46153; doi: 10.1038/srep46153 (2017).

**Publisher's note:** Springer Nature remains neutral with regard to jurisdictional claims in published maps and institutional affiliations.

## Supplementary Material

Supplementary Information
