# Supplementary material for: Suppressor of Cytokine Signaling 2 Negatively Regulates NK Cell Differentiation by Inhibiting JAK2 Activity
Source: Sci Rep. 2017 Apr 6;7:46153. doi: 10.1038/srep46153 (PMC5382670; doi:10.1038/srep46153)
Supplement: Supplementary Information [file srep46153-s1.pdf]

Supplementary information

Suppressor of Cytokine Signaling 2 Negatively Regulates NK Cell Differentiation by Inhibiting JAK2 Activity

Won Sam Kim,[1,2,5] Mi Jeong Kim,[1,2,5] Dong Oh Kim,[1,2] Jae-Eun Byun,[1,4] Hangsak Hui,[1,2] Hae Young Song,[1] Young-Jun Park,[1,2] Tae-Don Kim,[1,2] Suk Ran Yoon,[1,2] Eun-Ji Choi,[3] Haiyoung Jung[1,2,*] and Inpyo Choi[1,2,*]

Table S1. The frequency and absolute number of immune cells in WT or SOCS2$^{-/-}$ mice. The frequency and total cell numbers of immune cells are shown. The numbers of immune cells were determined by calculating the absolute number of each cell type from the FACS profiles. Results are expressed as mean ± SD of two separate experiments (n = 6 per group).

Figure S1. Engraftment of WT or SOCS2$^{-/-}$ HSCs in congenic recipients. (A,B) Engraftment of donor-derived HSCs in BM of recipient mice. **(C,D)** Engraftment of donor-derived HSCs in SP of recipient mice. Sorted HSCs of donor mice (CD45.2^{+}) were transplanted into lethally irradiated recipients (CD45.1^{+}). 4 months later, recipient mice were sacrificed and the donor-derived cell (CD45.2^{+}) frequency of the BM or SP was analyzed (n = 6 per group).

Figure S2. Expression of NK development markers. (**A-D**) WT or SOCS2$^{-/-}$ HPCs were differentiated with growth factors *in vitro*. NK cell precursors were cultured with IL-15 (30 ng/ml) and harvest at the indicated time points. Total cytoplasmic RNA was isolated, and the expression of SOCS2 (**A**), CD122 (**B**), and NK1.1 (**C**) genes was determined by RT-PCR at

the different stages of NK differentiation. (**D**) Cells were lysed and immunoblotted for the expression of ETS.1 and PU.1. A representative experiment of three is shown.

Figure S3. Expression of Pyk2 depending on IL-15 in mouse NK cells. (A, B) IL-15–primed primary WT and SOCS2$^{-/-}$ NK cells were cultured with IL-15 (10 ng/ml) or without IL-15 for 24 h. (**A**) The expression of p-Pyk2^{Tyr402}, Pyk2, p-JAK2, JAK2 and SOCS2 was determined by western blot analysis. (**B**) The levels of phospho-Pyk2 in primary NK cells of WT and SOCS2$^{-/-}$ mice were determined by confocal imaging with phospho-Pyk2 antibody. The data are representative of three independent experiments.

Figure S4. Distribution and migration of WT and SOCS2$^{-/-}$ NK cells in lung tissues. Lung tissues were digested three times by shaking for 30 min at 37 °C in RPMI 1640 medium containing 300 U/ml collagenase I (Worthington) and 20 ug/ml DNase I (Roche). Lung cells were then crushed through a 70-um nylon filter. (**A**) Representative FACS analysis pregated on lymphocytes from the lung of WT and SOCS2$^{-/-}$ mice. The numbers indicate the percentages of NK cells in the gated regions. (**B**) Bar graphs show the frequency and the numbers of NK cells in total lung cells. Data are shown as mean n = 5 mice per group. $^{*}p < 0.05$, $^{**}p < 0.01$ (error bars, mean ± SD). (**C-E**) After NK cell purification by FACSAria cell sorter (BD Biosciences), CD45.2^{+} NK cells (1×10^6 cells per mice) were i.v. injected into CD45.1^{+} recipients. On day 4 after i.v. injection, lungs were harvested, and the donor (CD45.2^{+}) and recipient (CD45.1^{+}) NK cells (NK1.1^{+}CD3^{-}) were quantified by flow cytometry (**C**). The percentage (**D**) and the numbers of NK cells (**E**) in total lung cells was determined by flow cytometry at the indicated group. Data are shown as mean n = 6 mice per group. $^{*}p < 0.05$, $^{**}p < 0.01$ (error bars, mean ± SD).

Table S1.

Table S1. The frequency and absolute number of immune cells in WT or SOCS2$^{-/-}$ mice.

	Bone marrow		Spleen	
	+/+	-/-	+/+	-/-
**frequency(%)**				
T cell	2.4 ± 0.5	2.5 ± 0.4	29.6 ± 1.8	28.0 ± 3.4
B cell	27.3 ± 5.6	29.6 ± 4.7	43.0 ± 4.3	41.7 ± 2.4
Gr1/Monocyte	36.3 ± 4.2	35.5 ± 5.4	1.6 ± 0.3	1.7 ± 0.4
**cell number**				
T cell (x10^5)	11.7 ± 2.6	12.7 ± 2.4	195.9 ± 22.7	193.0 ± 30.3
B cell (x10^6)	13.8 ± 2.8	15.3 ± 4.3	27.9 ± 6.8	28.5 ± 6.5
Gr1/Monocyte (x10^6)	16.6 ± 2.5	18.0 ± 3.7	1.05 ± 0.3	1.16 ± 0.3

Figure S1.

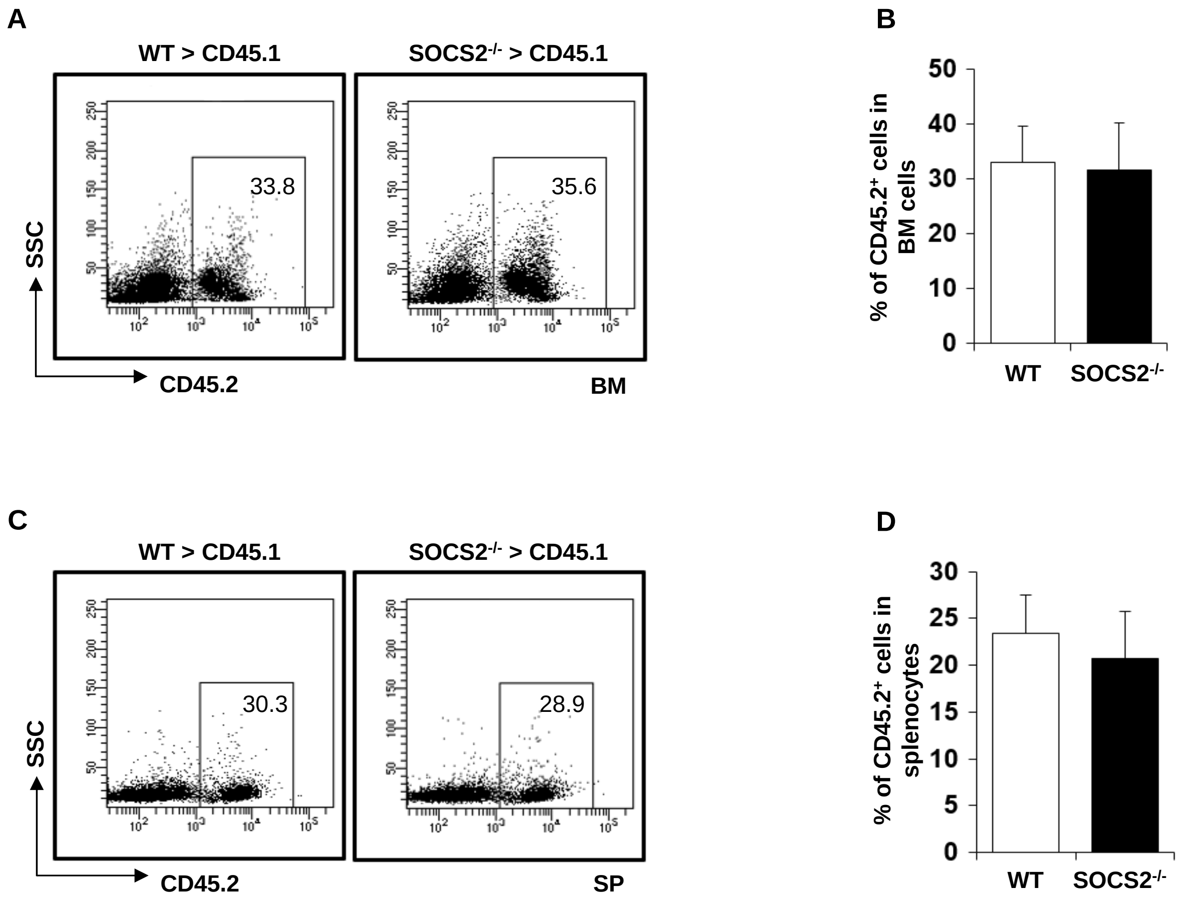

A
WT > CD45.1
SOCS2-/- > CD45.1
33.8
35.6
SSC
CD45.2
BM
B
% of CD45.2+ cells in BM cells
50
40
30
20
10
0
WT
SOCS2-/-
C
WT > CD45.1
SOCS2-/- > CD45.1
30.3
28.9
SSC
CD45.2
SP
D
% of CD45.2+ cells in splenocytes
30
25
20
15
10
5
0
WT
SOCS2-/-

Figure S2.

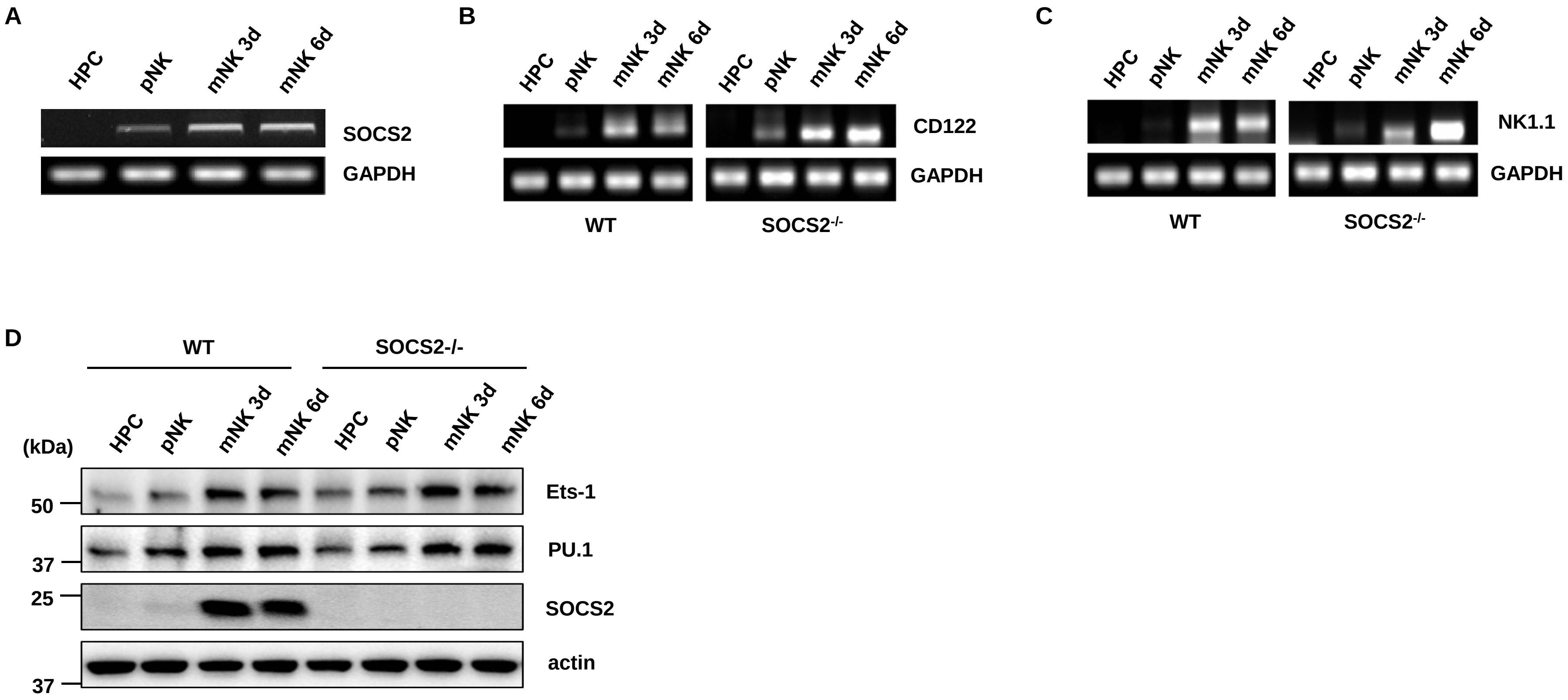
A
HPC
pNK
mNK 3d
mNK 6d
SOCS2
GAPDH
B
HPC
pNK
mNK 3d
mNK 6d
HPC
pNK
mNK 3d
mNK 6d
CD122
GAPDH
WT
SOCS2-/-
C
HPC
pNK
mNK 3d
mNK 6d
HPC
pNK
mNK 3d
mNK 6d
NK1.1
GAPDH
WT
SOCS2-/-
D
WT
SOCS2-/-
(kDa)
HPC
pNK
mNK 3d
mNK 6d
HPC
pNK
mNK 3d
mNK 6d
50
Ets-1
37
PU.1
25
SOCS2
37
actin

Figure S3.

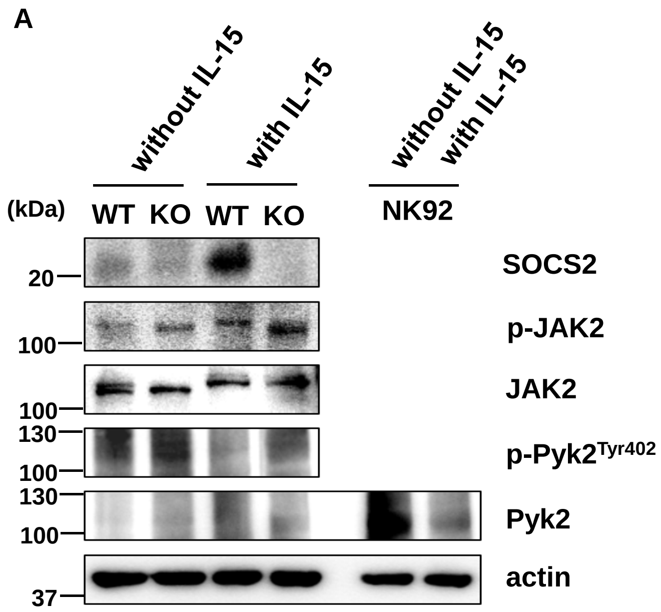

A
without IL-15
with IL-15
without IL-15
with IL-15
(kDa)
WT
KO
WT
KO
NK92
20
100
100
130
100
130
100
37
SOCS2
p-JAK2
JAK2
p-Pyk2Tyr402
Pyk2
actin

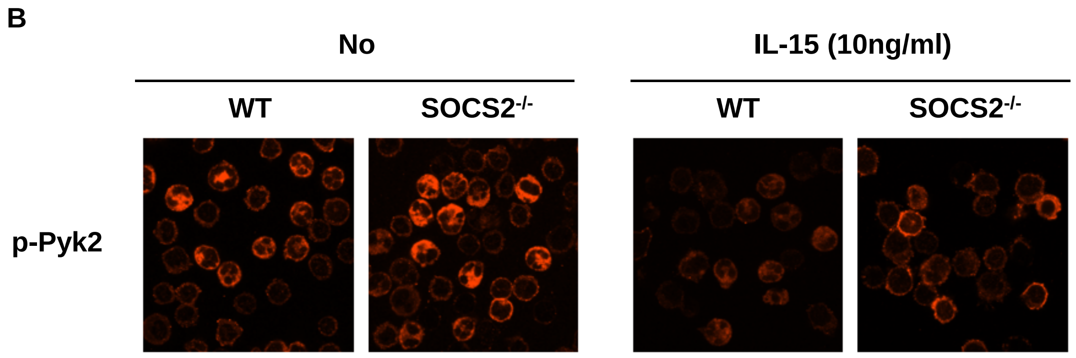

B
No
IL-15 (10ng/ml)
WT
SOCS2-/-
WT
SOCS2-/-
p-Pyk2

Figure S4.

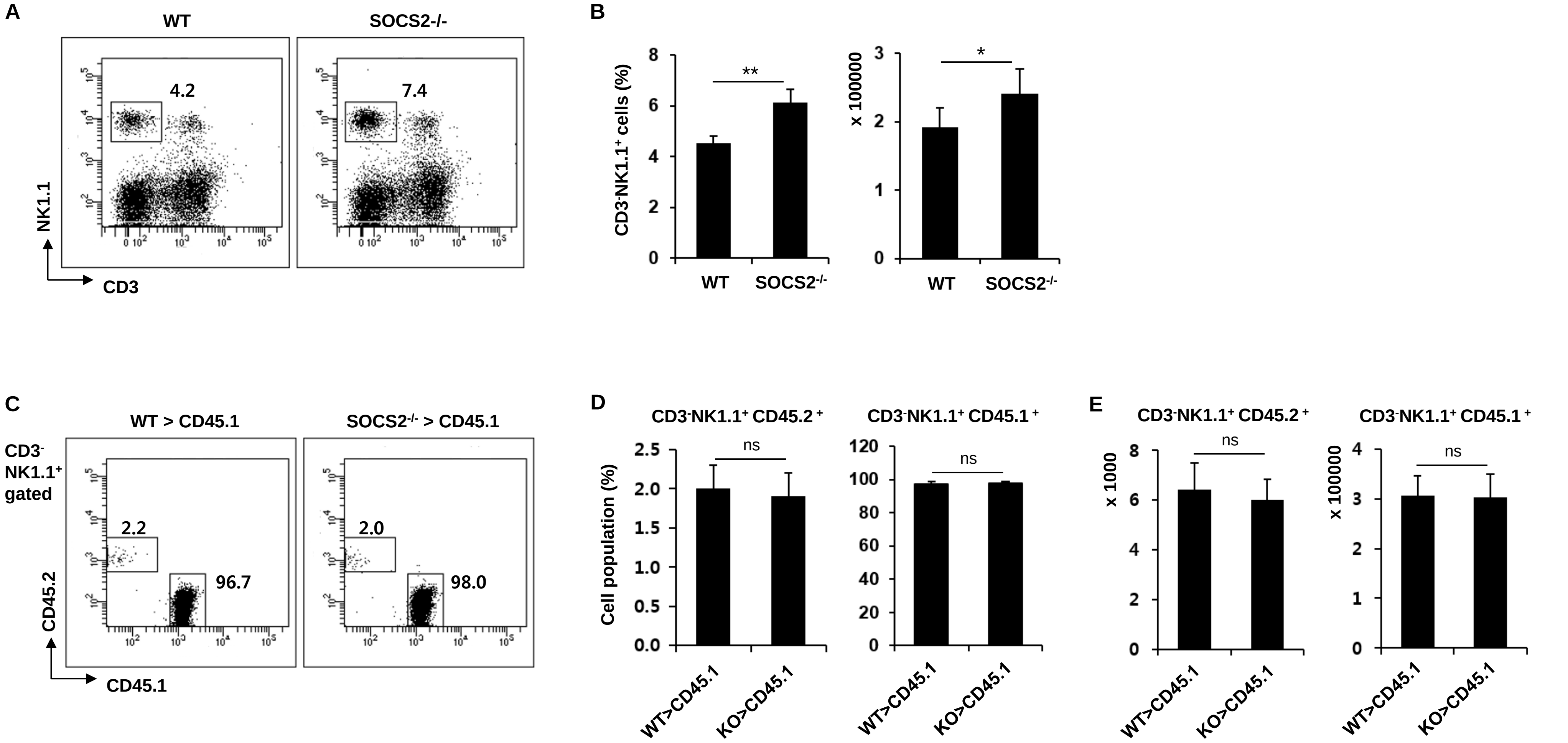
A
WT
SOCS2-/-
4.2
7.4
NK1.1
CD3
B
CD3-NK1.1+ cells (%)
**
WT
SOCS2-/-
x 100000
*
C
WT > CD45.1
SOCS2-/- > CD45.1
CD3- NK1.1+ gated
2.2
96.7
2.0
98.0
CD45.2
CD45.1
D
CD3-NK1.1+ CD45.2+
CD3-NK1.1+ CD45.1+
Cell population (%)
ns
WT>CD45.1
KO>CD45.1
E
x 1000
x 100000
